# Usefulness of argon plasma coagulation for bleeding around hepaticojejunal anastomosis

**DOI:** 10.1002/deo2.69

**Published:** 2021-11-02

**Authors:** Yoshitaka Tange, Naoyuki Hasegawa, Yutaro Sugiyama, Masato Endo, Masahiko Terasaki, Yoshiyuki Yamamoto, Kazunori Ishige, Kuniaki Fukuda, Hideo Suzuki, Yuji Mizokami

**Affiliations:** ^1^ Department of Gastroenterology Faculty of Medicine University of Tsukuba Ibaraki Japan; ^2^ Department of Gastroenterology National Hospital Organization Kasumigaura Medical Center Ibaraki Japan

**Keywords:** angiodysplasia, argon plasma coagulation, balloon enteroscopy, portal hypertension, surgical anastomosis

## Abstract

Ectopic varices due to extrahepatic portal vein obstruction (EHO) after hepaticojejunostomy have been previously reported. However, few case reports have described angiodysplasia‐like lesions due to EHO around the hepaticojejunal anastomosis because they comprise small vessels in the mucosal surface and cannot be detected by contrast‐enhanced computed tomography. Physicians need to insert the endoscope into the long afferent limb to diagnose angiodysplasia‐like lesions around the hepaticojejunal anastomosis. Some reports have described that endoscopy stops bleeding from angiodysplasia‐like lesions around the hepaticojejunal anastomosis; however, a standard methodology remains to be established. We present three cases of bleeding from an angiodysplasia‐like lesion around the hepaticojejunal anastomosis that were successfully treated using argon plasma coagulation (APC) with balloon‐assisted enteroscopy. Although one patient died owing to cancer progression 3 months after APC hemostasis, the hemostatic effect persisted for >2 years in the remaining two patients. These results suggest that APC is a good treatment option to stop bleeding from angiodysplasia‐like lesions at hepaticojejunal anastomosis.

## INTRODUCTION

Extrahepatic portal venous obstruction (EHO) after an abdominal operation is reportedly a cause of ectopic varices,^1^ and angiodysplasia‐like lesions have been reported to be a result of EHO.^2^, ^3^ Such varices and lesions tend to form near the anastomosis.^2^, ^3^, ^4^ The hepaticojejunal anastomosis is difficult to approach by conventional endoscopy because it is located in the long afferent limb. Balloon‐assisted enteroscopy was recently used to diagnose and treat bleeding from the hepaticojejunal anastomosis.^3^ Argon plasma coagulation (APC) and endoscopic clipping have been reported in cases in which they were used to stop bleeding from angiodysplasia‐like lesions at the anastomosis by endoscopy.^2^, ^3^ Here, we present three cases of bleeding from an angiodysplasia‐like lesion around the hepaticojejunal anastomosis that were treated using APC with balloon‐assisted enteroscopy. In all three cases, there were spider web like small veins with oozing around the hepaticojejunal anastomosis. We describe these findings as “angiodysplasia‐like lesion.” There is no matched classification in Yano‐Yamamoto classification Type1‐3. We need to classify our cases as Type 4 (unclassifiable) in Yano‐Yamamoto classification.^4^

This study was conducted in accordance with the ethical standards defined in the Declaration of Helsinki and was approved by the ethics committee of the University of Tsukuba (R03‐089). The requirement for informed consent was waived.

## CASE REPORT

### Case 1

**FIGURE 1:**
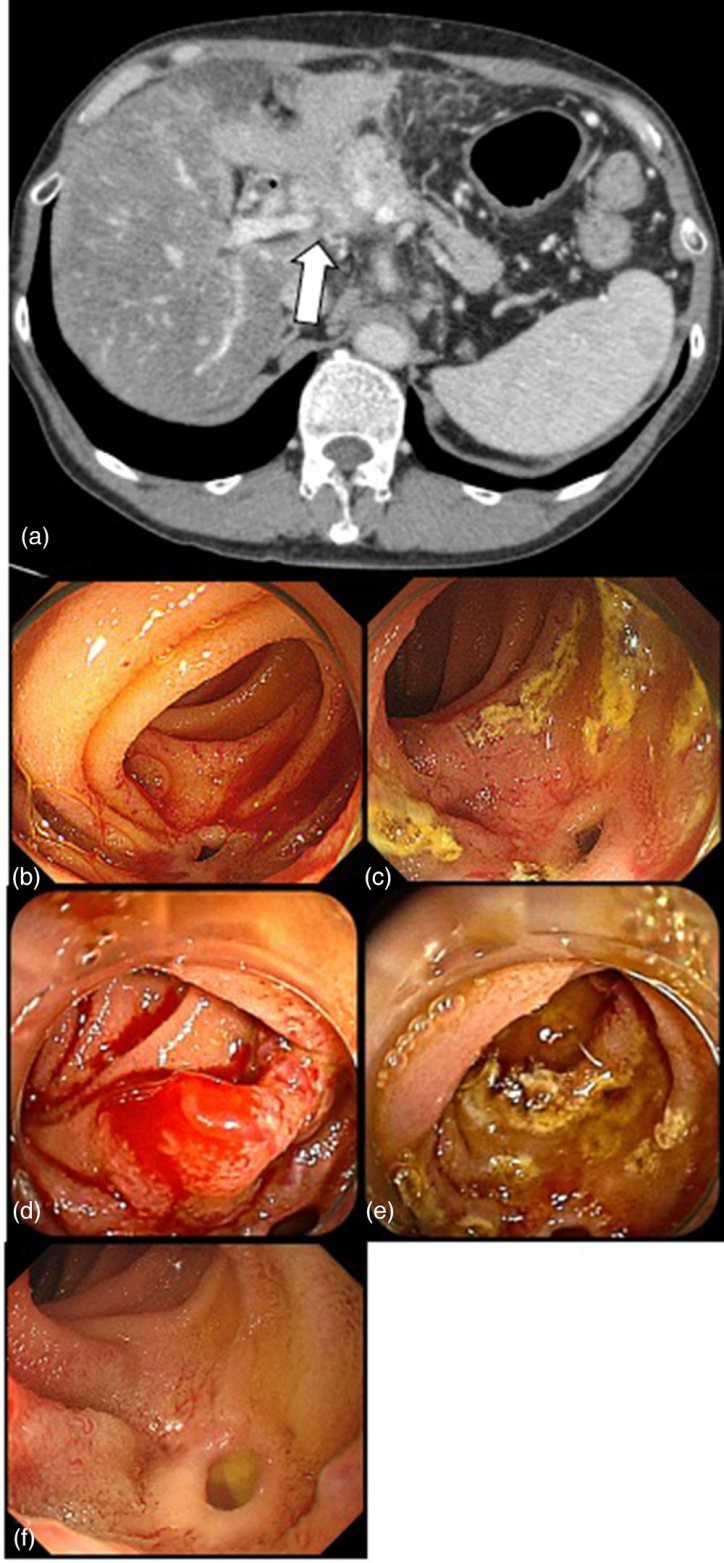
In case 1, contrast‐enhanced computed tomography showed portal vein stenosis (a). Endoscopic image shows the angiodysplasia‐like lesions around the hepaticojejunostomy and bleeding (b). We employed argon plasma coagulation (c). Three years after hemostasis, bleeding from the angiodysplasia‐like lesion around the hepaticojejunal anastomosis recurred (d). We employed argon plasma coagulation (e). Two years later, there was no further bleeding (f). White arrow: portal vein stenosis

A 64‐year‐old man presented with suspected cancer of the pancreatic head, which was identified by diagnostic imaging and treated with pancreatoduodenectomy (Figure 4a). Pathological examination led to the diagnosis of IgG4‐related disease. After the operation, portal vein stenosis progressed gradually owing to inflammation around the portal vein. Three years after the operation, esophageal varices (EV) appeared, and endoscopic variceal sclerotherapy (EIS) and endoscopic variceal ligation were performed. A year later, he was admitted to our hospital with melena. Contrast‐enhanced computed tomography showed that the portal vein stenosis worsened and extended to the superior mesenteric vein (Figure 1a). Bleeding from the EV was not observed on esophagogastroduodenoscopy (EGD), but we performed additional EIS because red color signs were observed on the EV surface. However, melena persisted after EIS and anemia gradually developed (Hb 7.3g/dl). We then examined the afferent limb using conventional upper gastrointestinal endoscopy (GIF‐Q260J; Olympus, Tokyo, Japan). We successfully inserted an endoscope into deep inside the afferent limb and found the angiodysplasia‐like lesions around the hepaticojejunal anastomosis. The lesions bled easily, and we employed APC (40 W, flow 1 L/min, ICC200, ERBE, Tuebingen, Germany) (Figure 1b,c). After APC, melena and anemia improved, and the patient was discharged. Three years after APC, bleeding from the angiodysplasia‐like lesion around the hepaticojejunal anastomosis recurred, and we performed APC to stop the bleeding (Figure 1d,e). At that time, we could not insert a conventional upper gastrointestinal endoscope into deep inside the afferent limb; therefore, we used balloon‐assisted enteroscopy (EI‐530B; FUJIFILM, Tokyo, Japan). There was no further bleeding for 2 years after the second APC (Figure 1f).

### Case 2

**FIGURE 2:**
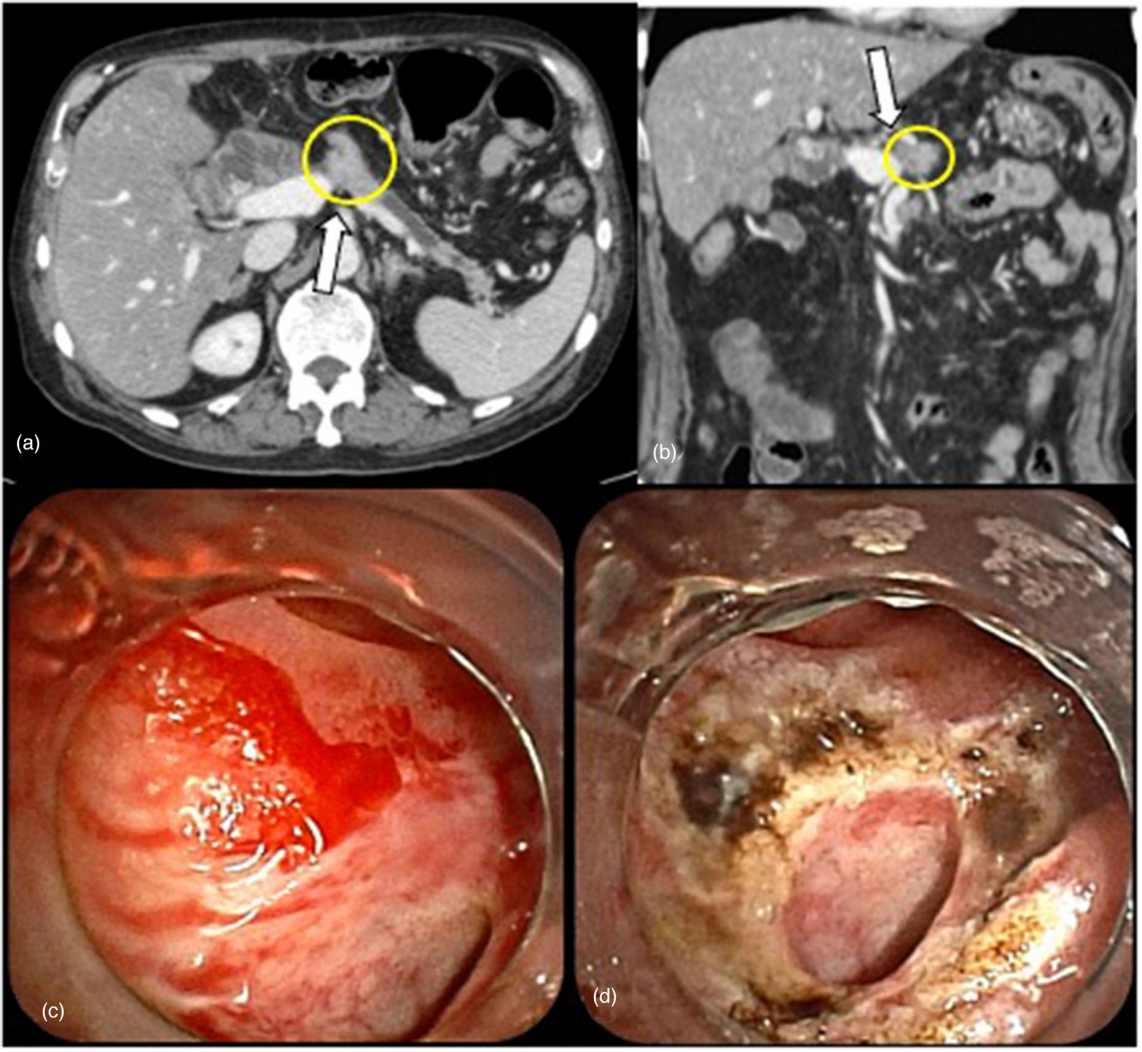
In case 2, contrast‐enhanced computed tomography showed portal vein stenosis due to recurrent tumor ((a) axial view, (b) coronal view). Endoscopic image shows the angiodysplasia‐like lesions around the hepaticojejunal anastomosis (c), and we employed argon plasma coagulation (d). White arrow: portal vein stenosis, yellow circle: recurrent tumor

A 74‐year‐old man had undergone pancreaticoduodenectomy for pancreatic cancer (Figure 4a). At 5 months after the operation, he experienced local recurrence, and S‐1 monotherapy was initiated. However, 3 months later, multiple lung and bone metastases appeared, and gemcitabine plus nab‐paclitaxel therapy was initiated. At 1 year and 4 months after the operation, he was admitted to our hospital with melena and severe anemia (Hb 6.3 g/dl). He was subjected to EGD, colonoscopy, and capsule endoscopy, none of which revealed the cause of bleeding. Contrast‐enhanced computed tomography showed portal vein stenosis due to the recurrent tumor and collateral vessels around the hepaticojejunal anastomosis (Figure 2a,b). We performed balloon‐assisted enteroscopy, which revealed the angiodysplasia‐like lesions around the hepaticojejunal anastomosis. The lesions bled easily and were treated with APC (Figure 2c,d). After APC, he was discharged without progressive anemia; however, he experienced rebleeding 3 months later, and APC was performed with balloon‐assisted enteroscopy. Subsequently, although no rebleeding occurred, he died 3 months after the final hemostasis owing to cancer progression.

### Case 3

**FIGURE 3:**
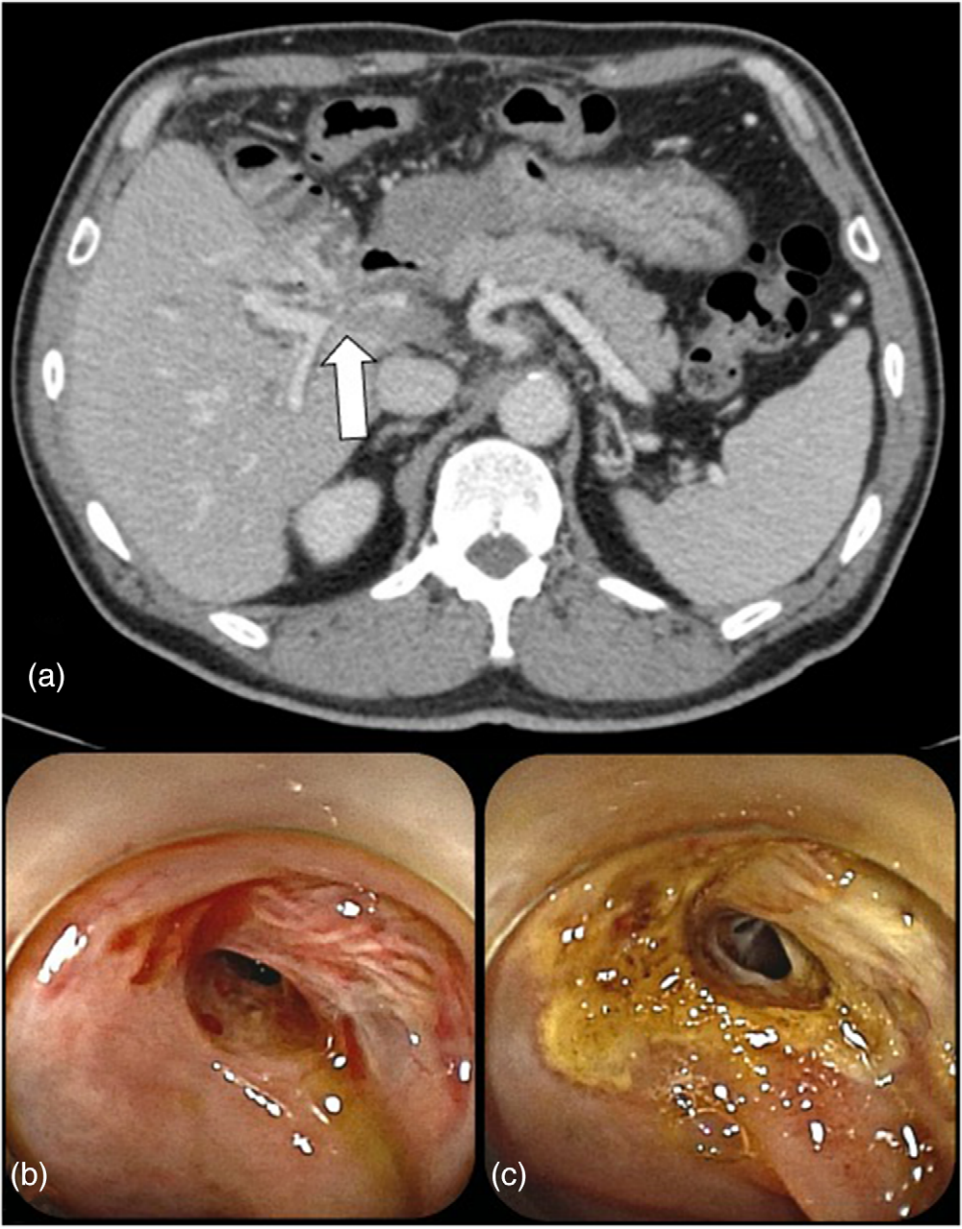
In case 3, contrast‐enhanced computed tomography showed portal vein stenosis (a). Endoscopic image shows the angiodysplasia‐like lesions around the hepaticojejunal anastomosis (b), and we employed argon plasma coagulation (c). White arrow: portal vein stenosis

**FIGURE 4:**
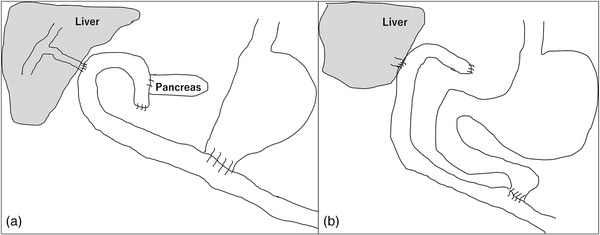
(a) Illustration of intestinal reconstruction after pancreatoduodenectomy. (b) Illustration of intestinal reconstruction after Roux‐en‐Y hepaticojejunostomy

A 55‐year‐old man had undergone extended left hepatectomy and bile duct resection with Roux‐en‐Y hepaticojejunostomy for intrahepatic cholangiocarcinoma (Figure 4b). Four years after the operation, he experienced local recurrence in the liver, and gemcitabine and proton beam therapy were initiated. A month after initiating proton beam therapy, he was admitted to our hospital with melena and severe anemia (Hb 7.1 g/dl). Contrast‐enhanced computed tomography revealed portal vein stenosis (Figure 3a). Balloon‐assisted enteroscopy revealed angiodysplasia‐like lesions around the hepaticojejunal anastomosis, and the lesions bled easily. We employed APC to stop the bleeding (Figure 3b,c). After APC, melena and anemia improved, and he was discharged. No further bleeding occurred for >3 years.

## DISCUSSION

Ectopic varices and angiodysplasia‐like lesions at hepaticojejunal anastomosis due to EHO have been reported in several previous studies.^1^, ^2^, ^3^, ^5^, ^6^, ^7^, ^8^ Endoscopic treatment (such as APC^3^ and endoscopic clipping^2^) or interventional radiology (such as portal vein stenting^6^, ^7^ and transjugular intrahepatic portosystemic shunt^8^) has been performed to stop bleeding from the ectopic varices or angiodysplasia‐like lesions at hepaticojejunal anastomosis. However, angiodysplasia‐like lesions at the anastomosis cannot be detected on computed tomography; therefore, there have been few reported cases, and no effective treatment has been found to date. APC has been widely employed to treat bleeding from small bowel angiodysplasia‐like lesions.^9^ APC procedures are simple and provide quick coagulation. APC triggers coagulation only at the mucosal surface, and its safety in the small bowel has been established.^9^, ^10^ However, the use of APC should generally be treated with great caution in the jejunum because of the small space and sparse submucosa of the jejunum. Moreover, the use of APC at hepaticojejunal anastomosis may increase the risk of cholangitis if argon gas is not aspirated in moderation. To ensure safety, we limited the APC power setting and flow rate setting to around 40 W and 1 L/min respectively. These settings are lower than the general setting for radiation proctitis (40–60 W, 0.6–3.0 L/min).^10^ Also, limitation of APC flow rate setting reduces the risk of argon gas embolism. Additionally, we used carbon dioxide (CO2) insufflation during balloon‐assisted enteroscopy to prevent air embolization. Neumann et al. reported one case in which angiodysplasia‐like lesions at hepaticojejunal anastomosis were successfully treated using APC.^3^ In their case, no rebleeding observed for 2.5 months after APC hemostasis; however, there have been no reports on long‐term outcomes after APC hemostasis. Here, we reported three cases in which APC hemostasis was successfully employed to treat angiodysplasia‐like lesions around the hepaticojejunal anastomosis. This is the first report to describe multiple cases of APC and long‐term prognosis.

In this study, case 1 had no rebleeding for 3 years after the first APC hemostasis. At the time of rebleeding, APC hemostasis was performed again, and no rebleeding was observed for the next 2 years. In case 2, APC hemostasis was performed for rebleeding, and bleeding was controlled. In case 3, rebleeding was not observed for 3 years after APC hemostasis. These results suggest that APC maintains the hemostatic effect for an extended period, and even if rebleeding is observed, APC can be repeated safely and effectively.

Balloon‐assisted enteroscopy helps diagnose and treat angiodysplasia‐like lesions around the hepaticojejunal anastomosis, and this method is likely to gain broad adoption. The combination of balloon‐assisted enteroscopy and APC is suitable for treating bleeding from angiodysplasia‐like lesions around the hepaticojejunal anastomosis.

There are some limitations in our report. First, there were only three patients who received APC hemostasis. Second, our study is retrospective. Prospective studies with a large cohort are needed to evaluate the efficacy of APC hemostasis.

In conclusion, APC is a promising treatment for patients who experience refractory bleeding due to angiodysplasia‐like lesions at hepaticojejunal anastomosis.

## CONFLICT OF INTEREST

The authors declare no conflict of interest for this article.

## FUNDING INFORMATION

None.
